# Prospects of Nanostructure Materials and Their Composites as Antimicrobial Agents

**DOI:** 10.3389/fmicb.2018.00422

**Published:** 2018-03-09

**Authors:** Anupriya Baranwal, Ananya Srivastava, Pradeep Kumar, Vivek K. Bajpai, Pawan K. Maurya, Pranjal Chandra

**Affiliations:** ^1^Department of Biosciences and Bioengineering, Indian Institute of Technology Guwahati, Guwahati, India; ^2^Department of Pharmacology and Toxicology, National Institute of Pharmaceutical Education and Research, Guwahati, India; ^3^Department of Forestry, North Eastern Regional Institute of Science and Technology, Deemed University, Nirjuli, India; ^4^Department of Energy and Materials Engineering, Dongguk University-Seoul, Seoul, South Korea; ^5^Interdisciplinary Laboratory of Clinical Neuroscience (LiNC), Department of Psychiatry, Universidade Federal de São Paulo-UNIFESP, São Paulo, Brazil

**Keywords:** nanostructured material, antimicrobial activity, cytotoxicity, human health, antimicrobial agent

## Abstract

Nanostructured materials (NSMs) have increasingly been used as a substitute for antibiotics and additives in various products to impart microbicidal effect. In particular, use of silver nanoparticles (AgNPs) has garnered huge researchers' attention as potent bactericidal agent due to the inherent antimicrobial property of the silver metal. Moreover, other nanomaterials (carbon nanotubes, fullerenes, graphene, chitosan, etc.) have also been studied for their antimicrobial effects in order ensure their application in widespread domains. The present review exclusively emphasizes on materials that possess antimicrobial activity in nanoscale range and describes their various modes of antimicrobial action. It also entails broad classification of NSMs along with their application in various fields. For instance, use of AgNPs in consumer products, gold nanoparticles (AuNPs) in drug delivery. Likewise, use of zinc oxide nanoparticles (ZnO-NPs) and titanium dioxide nanoparticles (TiO_2_-NPs) as additives in consumer merchandises and nanoscale chitosan (NCH) in medical products and wastewater treatment. Furthermore, this review briefly discusses the current scenario of antimicrobial nanostructured materials (aNSMs), limitations of current research and their future prospects. To put various perceptive insights on the recent advancements of such antimicrobials, an extended table is incorporated, which describes effect of NSMs of different dimensions on test microorganisms along with their potential widespread applications.

## Introduction

Microbial contamination even today is amongst primal causes of morbidity and mortality across the globe. According to reports, about half of the population in developing countries are infested with microbial contamination and annually more than 3 million people die because of it (Armentano et al., 2014). Despite spectacular advances in diagnostic and therapeutic strategies, microbial infections continue to affect biomedical and healthcare sectors due to the emergence of resistance against several available antibiotics (Murphy, 1994; Desselberger, 2000). Numerous factors including but not limited to human lifestyle changes, industrialization, civil wars, and microbial genome alterations have been recognized for their involvement in emergence or re-emergence of pathogens (Morse, 2001). Keeping this serious issue in consideration, development of better antimicrobial drugs has become highly imperative. Other than aforementioned issue, microbes are also known for deteriorating textiles, spoiling food products, contaminating surgical instruments and causing the damage to crops. The available conventional solutions to avert these problems are not sufficient enough, therefore, development of better alternatives is highly sought to secure the basic living standard of human beings.

Recent advances in nanostructure-based antimicrobial medications have unveiled novel prospects to combat drug resistance in microbes. Therefore, usage of NSM as an antimicrobial agent in both particle and composite form has gained enormous importance in recent years. Application of NSM in biomedical domain relies on a number of unique properties *viz*. optical, physical, chemical, thermal, electrical, etc. Some of these unique properties play a crucial role in providing medical relevance to the NSM while, the other properties enable them to have significance in other industries (Dakal et al., 2016). The pivotal characteristics that an aNSM should preferably possess are broad-spectrum effect, inexpensive, high specificity, and least or negligible susceptibility toward resistance development (Beyth et al., 2015). Both inorganic and organic NSMs have shown antimicrobial effects over a wide range of microbial strains (Dastjerdi and Montazer, 2010; Li et al., 2011; Latif et al., 2015), paving way for their potential applications in textile industry (Dastjerdi and Montazer, 2010), food packaging and processing industry (Duncan, 2011), agricultural products and crop safety (Khot et al., 2012), water treatment (Li et al., 2008), and construction industry (Lee et al., 2010) to prevent damages associated with microbial growth.

In this review, we have presented a broad classification of NSMs produced via. different synthetic approaches along with an overview of the nanomaterials which possess antimicrobial activity. Though, it is practically impossible to present a comprehensive overview on all NSMs including their method of synthesis, characterization techniques, and mode of antimicrobial activity in this review. However, we have tried to present a report which clearly heralds the current scenario of application of aNSMs in widespread domains along with inadequacies of current research and future prospects of NSMs as antimicrobial agents.

## Classification of nanostructured materials

**Figure 1:**
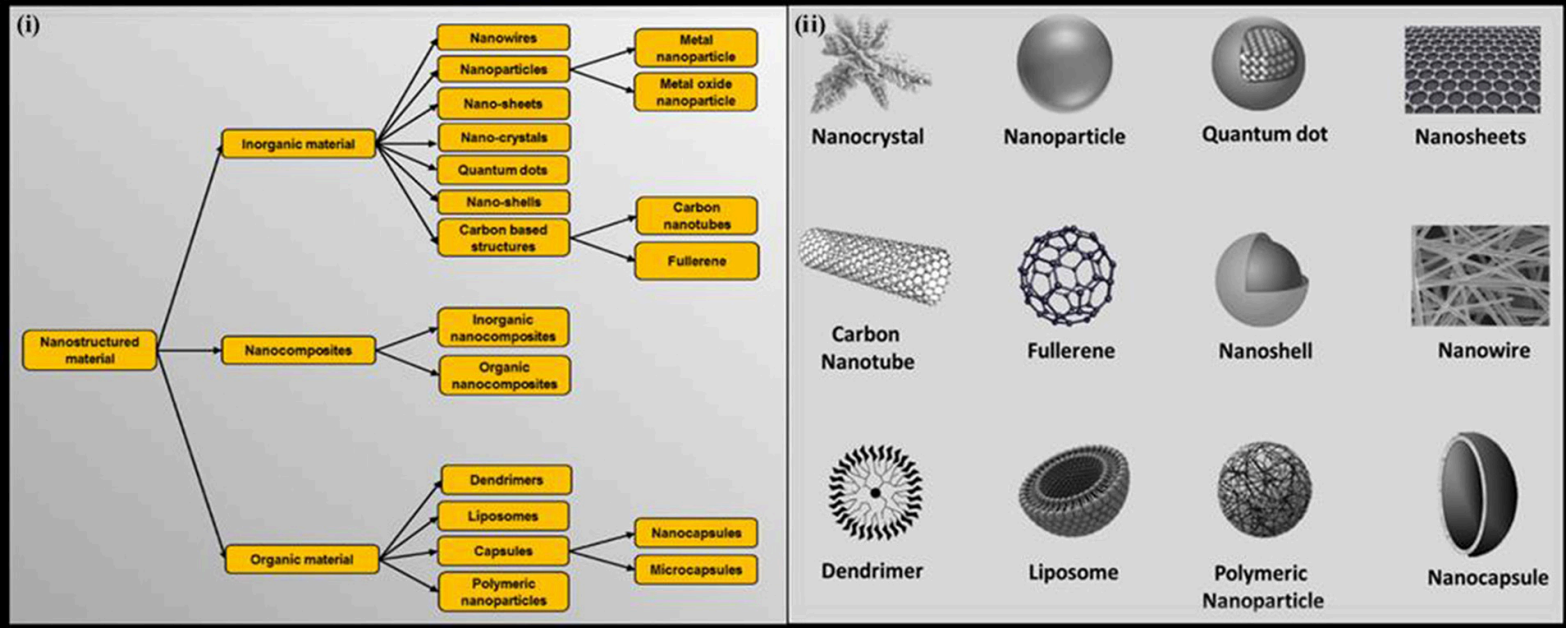
**(i)** Illustration representing classification of nanostructured materials used as antimicrobials and **(ii)** depiction of various forms of nanostructured materials and their morphology.

A wide variety of materials exist today that is colloquially considered as NSMs, but the term NSM validates only those materials which belong to 1–100 nm range. NSMs may exhibit large particle size (>100 nm) when they combine with other materials (like polymers, biomolecules, other NSMs, etc.) to form composite NSM or when they exist in the form of aggregates (Bhushan, 2010). NSMs are broadly classified into three categories, which are further classified into different sub-categories (Figure 1i). The inorganic NSMs include nanosheets (a 2-D nanostructure whose thickness lies in the nano range), metal and metal oxide nanoparticle (particles whose diameter is usually <100 nm), nanoshells (typically, spherical nanoparticles with a dielectric core enclosed inside thin metallic shell), nanowires (wire exhibiting diameter/thickness of few nanometers), nanocrystals (material composed of atoms aligned in single- or poly-crystalline arrangement with its one dimension usually <100 nm), quantum dots (3-D nanocrystals composed of semiconducting material with their diameter lying in 2–10 nm range), and carbon nanotubes (cylindrical carbon nanostructures with unusual properties). Organic NSMs comprise of dendrimers (3-D, hyperbranched, tree-like polymeric nanostructures), liposomes (nano-vesicles obtained from hydration of dry phospholipids), and nano/micro capsules (material composed of natural or synthetic polymer shells in order to enclose different active materials, such as drugs, catalysts, biomolecules, etc. as its core) (Dastjerdi and Montazer, 2010). These organic NSMs usually act as a carrier of inorganic nanoparticles and provide a wide range of biomedical applications. Figure 1ii shows morphological features of NSMs which exist in varied forms.

Various synthetic approaches are available today that can be employed to generate NSMs. Conventional synthetic approaches include physical and chemical processes; however, biological processes are one of the newer approaches to allow synthesis of NSMs. In certain cases, especially invasive biomedical application of NSMs, biological approaches (microbe or plant extract assisted) become a method of choice over other conventional approaches because these (biological) approaches do not elicit cellular toxicity (Ahmed et al., 2016; Baranwal et al., 2016).

## Antimicrobial nanostructured materials

**Figure 2:**
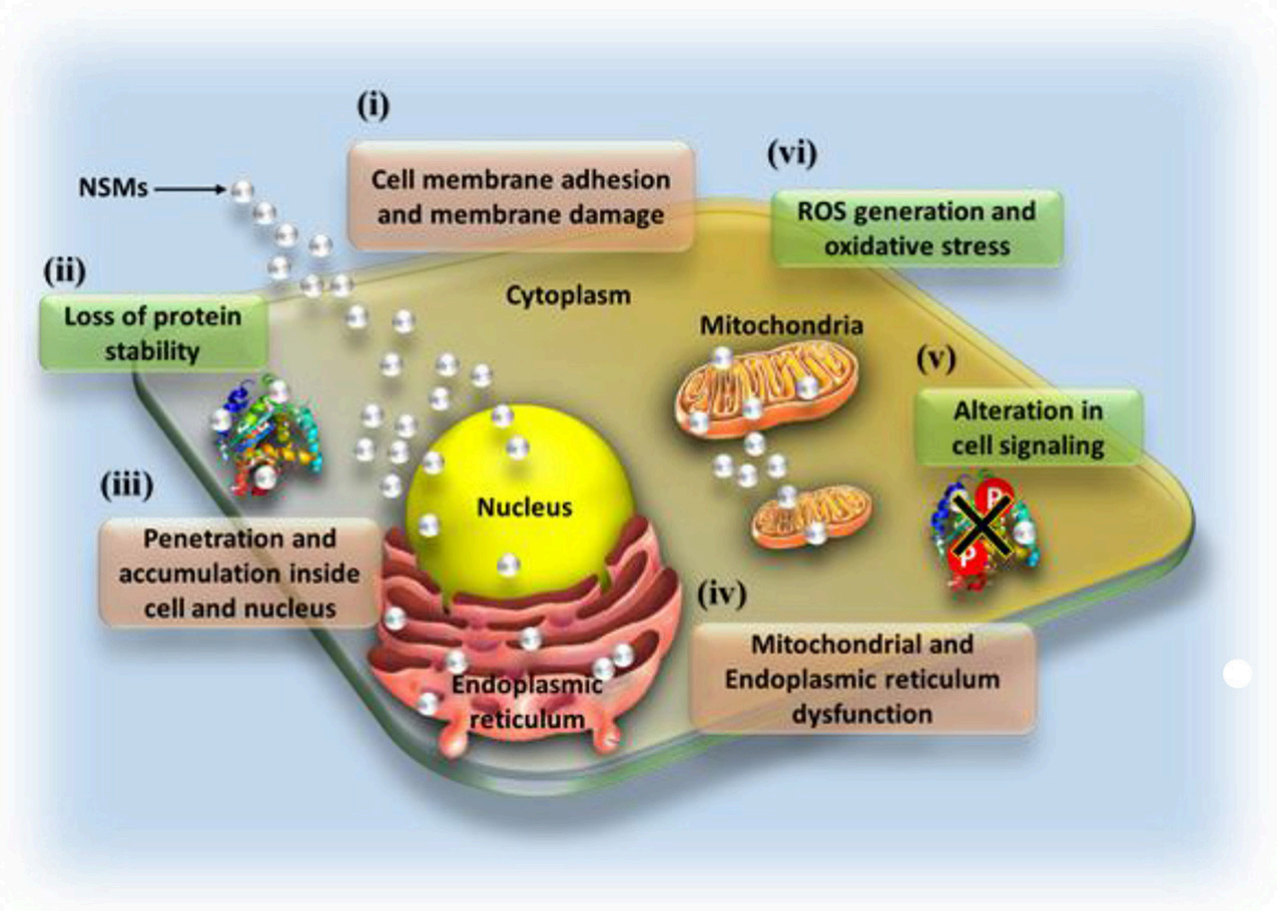
Various modes of microbial toxicity caused by nanostructured materials.

Antimicrobial effect of NSMs has been widely studied by several research groups against a wide range of microorganisms. NSMs can be regarded as the next generation antibiotics as they possess remarkable potential to overcome multidrug resistance problems in the pathogenic microbes. Depending on their ability to provide biostatic and biocidal action against microbial species, they can also be exploited in healthcare and personal care products, food safety, crop protection, water treatment, textile industries, etc. Although NSMs have shown spectacular antimicrobial effect against more than 500 microbial species, however, accurate mechanism behind their microbicidal activity is not hitherto well-understood (Beyth et al., 2015). Nonetheless, some widely accepted modes of mechanisms of antimicrobial action (Figure 2) are discussed in the following section.

## Mechanisms of microbial toxicity

Electrostatic attraction between cationic NSM and anionic microbial cell membrane instigates adhesion of NSMs onto the cell wall or cell membrane which leads to cytosol shrinkage and detachment of the membrane, and eventually cell wall rupture (Dakal et al., 2016). Adhesion of NSM in some cases is followed by its penetration across the cell membrane where, it binds with biomolecules (DNA, protein, and lipids) and cause damage to them which thus, hamper crucial pathways and result in microbial cell death (Li et al., 2008). Apart from electrostatic attraction, the interaction of sulfur groups present in cell wall proteins and NSM leads to irreversible changes in the cell wall structure which subsequently disturbs the lipid bilayer integrity and increases the membrane permeability (Ghosh et al., 2012). Further modes of antimicrobial action of NSM involve the formation of reactive oxygen species (ROS) which cause increase in oxidative stress inside microbial cells. The increased levels of ROS and other free radicals result in mitochondrial and endoplasmic reticulum dysfunction and irreversible damage to biomolecules that subsequently cause genotoxic effects (Huang et al., 2008; Dizaj et al., 2014). NSMs, especially nanoparticles modulate microbial signal transduction pathways by causing de-phosphorylation of tyrosine residues on crucial proteins and thus, impart their antimicrobial effect (Dakal et al., 2016).

## Current scenario of antimicrobial nanostructured materials' applications

A wide range of NSMs, such as metal and metal oxide nanoparticles, NCH, carbon nanotubes (CNTs), organic nanoparticles (ONPs), etc. have found their usage in widespread domains of consumer products, food safety, agricultural products, crop protection, and industrial processes (waste water treatment, architectural/construction material, etc.). Such examples of NSMs along with their dimension analysis, antimicrobial effect on test microorganisms, and potential applications thereof has been discussed comprehensively in Table 1. This table has been complied by including reports published between year 2007 and 2018 explicitly. Though there are several reports of NSMs being used in commercial products, however, their exact nano-formulation is not disclosed anywhere, most likely due to trade-secret constraints. Some of the commercial examples of aNSM based products are nasiol® AntiMoss protection, nasiol® HomeWood protection (https://nasiolgulf.com/), I-canNano metal paints, and I-canNano fillers (https://www.icannanopaints.com/), NanoSeal™ NanoPack (Duncan, 2011), 4Care Lenscare nano-Behälter, Acticoat Antibacterial barrier, JR Nanotech SoleFreshT nanosilver socks, and Miradent Miradent gelée toothpaste and mouth wash (Wijnhoven et al., 2010). Following section exclusively deals with applications of aNSMs in aforementioned domains.

**Table 1:** Different nanostructured materials and composites with their antimicrobial effect against selected strains and potential applications in different fields.

**Nanostructured materials and composites**	**Size/diameter (nm)**	**Test microbial organisms**	**Effect of nanostructured material**	**Potential industrial applications**	**References**
ZnO nano needle	ca. 63	*Escherichia coli, Bacillus subtilis*, and *Aspergillus niger*	Successful inhibition of test microbes was observed	Functional building material	Singh et al., 2018
Nano-liposomal formulation of mupirocin	*NR*	*Neisseria gonorrhoeae*	Highly efficacious antibacterial activity was observed	Next generation antibiotics	Cern et al., 2018
Chitosan (CS) functionalized polyaniline-polypyrrole copolymer	200	*E. coli and E. agglomerans*	Excellent antimicrobial activity against bacterial strains	Biomedical devices, water filters, and instrument preparation	Kumar et al., 2017
Graphene oxide-chitosan (CS-GO) nanocomposite	*NR*	*E. coli and B. subtilis*	Efficient bacterial inactivation was observed	Food packaging	Grande et al., 2017
Polypyrole/Cu-doped ZnO nanocomposite	*24*	*E. coli and B. subtilis*	Successful inhibition of test microbes was observed	Environmental pollution monitoring	Khan et al., 2017
ZnO-NP coated cotton composites	8–12	*E. coli, S. aureus, C. albicans, and Microsporum canis*	Successful inhibition of test microbes was observed	Textile industry	El-Nahhal et al., 2017
Fe_2_O_3_-NPs	*24*	*Bacillus cereus and Klebsiella pneumonia*	High antibacterial activity was evident	Antimicrobial and biomedical applications	Ansari et al., 2017
AgNPs	20–30	*E. coli, B. subtilis, S. cerevisiae, and C. albicans*	Highest sensitivity was evident for *E. coli, S. cerevisiae, and C. albicans*	Textile industry	Khatoon et al., 2017
ZnO-ZnS@polyaniline nanocomposite	*NR*	*E. coli*	High antibacterial activity was evident	Waste water treatment	Anjum et al., 2017
AgNPs	20–30	*E. coli and S. aureus*	Diminished bacterial growth was evident	Portable water filters, medical devices, food packaging, clothing, washing machine and refrigerator coating, and storage containers	Andrade et al., 2016
AgNPs	13.56–18.33	*Candida albicans*	Successful inhibition of growth of *C. albicans*	Antifungal medication against urinary tract infection (UTI)	Oves et al., 2016
Hydroxyapatite—AgNP composite	*NR*	*E. coli and S. aureus*	Effective inhibition of bacterial strains even at low concentrations of AgNPs	Medical implants and dental applications	Andrade et al., 2016
Cobalt doped ZnO-NP	20.5–25.7	*Shigella dysenteriae, Salmonella typhi, Vibrio cholerae and E. coli*	Effective bactericidal effect against *Vibrio cholerae and E. coli was observed*	Waste water treatment	Oves et al., 2015
PEGylated Ag- Graphene quantum dots (GQDs) nanocomposite	*NR*	*P. aeruginosa* and *S. aureus*	Synergistic antibacterial effect of AgNP and GQD was observed	Next generation antibiotics	Habiba et al., 2015
AuNP stabilized liposome	*NR*	*S. aureus*	Successful antibacterial action was evident	Antibacterial agent and Drug delivery	Gao et al., 2014
(GQD)	20–67	Methicillin-resistant *S. aureus and E. coli*	Selective antibacterial photodynamic effect of GQD was evident	Next generation antibiotics	Ristic et al., 2014
AgNP-graphene oxide (GO) Nano-sheets composite	2–25	*S. aureus* and *B. subtilis*	Nanocomposite resulted in complete loss of bacterial stains	Next generation antibiotics	Das et al., 2013
AuNPs	45–75	*Puccinia graminis tritci, A. flavus, A. niger* and *C. albicans*	Effective inhibition of test fungal strains was evident	Antifungal medication	Jayaseelan et al., 2013
AgNPs wrapped in carbon (GO) nano-scrolls (composite)	30–50	*C. albicans* and *C. tropicalis*	Prolonged and enhanced antifungal activity was evident for nano-scrolls	Next generation antibiotics, medical, and health care products	Li et al., 2013
CuNP	2–350	*C. albicans*	Strong antifungal activity was evident	Dental materials	Usman et al., 2013
ZnO-NP	25 and 40	*S. aureus, S. marcescens*, and *P. mirabilis*	Prominent inhibition of the bacterial strains	Antimicrobial creams, lotions and ointments, sunscreen lotions, deodorants, ceramics, and self-cleaning glass	Gunalan et al., 2012
CuO-NP	20–21	*E. coli, P. aeruginosa, B. subtilis*, and *S. aureus*	Effective inhibition of test bacterial strains was evident	Next generation antibiotics	Azam et al., 2012
TiO_2_-NP/ZnO nano-wire nanocomposite	50–100	*C. albicans*	TiO_2_-NP assisted in providing enhanced antifungal activity of ZnO nano-wire	Next generation antifungal agent	Haghighi et al., 2011
CNT doped TiO_2_ thin film (nanocomposite)	5–30	*E. coli*	Photo-inactivation of the test organism was seen	Solar disinfection systems, antimicrobial surface coatings, anti- biofouling membranes, and wastewater treatment	Akhavan et al., 2010
iPP/TiO_2_/Ag nanocomposites	*NR*	Bacterial spp.	High percentage of biostatic efficiency was observed	Textile industry	Dastjerdi et al., 2010
AuNPs	22–52	*E. coli and S. aureus*	High antibacterial activity was observed	Next generation antibiotics	Rai et al., 2010
Zinc oxide quantum dots (Zn-QD)	5	*L. monocytogenes, E. coli*, and *S. enteritidis*	Dose dependent antibacterial activity was observed	Wound dressings	Jin et al., 2009
Nanodarts and SWNT	0.83 and 5–50	*E. coli, B. subtilis, P. aeruginosa*, and *S. aureus*	Nanodarts showed strong antibacterial activity than SWNT	Medical devices, anti-biofouling membranes, and wastewater treatment	Liu et al., 2009
CuO-NPs	20–95	Meticillin-resistant *S. aureus, S. epidermidis, Proteus* spp., *E. coli*, and *P. aeruginosa*	CuO-NPs successfully inhibited growth of test bacterial strains including Meticillin-resistant *S. aureus*	Next generation antibiotics, biosensing	Ren et al., 2009
AgNPs stabilized in highly branched polymer	1.4–7.1	*A. niger*	Prominent antifungal effect was evident	Bone cement	Zhang et al., 2008
AuNPs stabilized in hyper branched polymer	7.7–3.9	*B. subtilis, E. coli*, and *K. mobilis*	Considerable antibacterial activity was observed	Antibiotic drug delivery system	Zhang et al., 2008
PAMAM dendrimer	*NR*	*S. aureus* and *P. aeruginosa*	Better inhibition of *P. aeruginosa* was evident	Antibacterial agent and antibiotic drug delivery system	Calabretta et al., 2007
SWNT	0.9	*E. coli*	Strong antibacterial effect was observed	Water disinfection, architectural material, anti- biofouling membranes, and wastewater treatment	Kang et al., 2007
Nanofibers with embedded AgNPs	200–550	*E. coli* and *P. aeruginosa*	Enhanced antibacterial effect was evident in case of functionalized PAN nanofibers	Water treatment, medical, and health care products	Lala et al., 2007

*NR. not reported*.

### Metal/metal oxide nanoparticles

Amongst different types of metal nanoparticles (MNPs), AgNPs have witnessed their usage at much wider scale. Currently, they have been used in more than 100 consumer products for imparting antimicrobial effect, starting from storage wares, textiles, nutritional additives to kitchen appliance surface coatings, hospital consumables and wares, etc. (Li et al., 2008). The mechanism behind their microbicidal action is mostly accredited to release of Ag^+^ ions, cell membrane or cell wall damage, disruption of electron transport and signal transduction pathway, and damage to cellular DNA and proteins due to ROS (Dakal et al., 2016; Qayyum et al., 2017). AuNPs are one of the most valuable antibacterial agents due to their biocompatibility, higher potential of functionalization, and ease of detection. The mechanism behind antibacterial effect of AuNP is not yet fully explored; however, there have been reports of bacterial damage due to modification in membrane potential, loss of ATPs (Cui et al., 2012; Abdel-Raouf et al., 2017), and ROS generation (Zheng et al., 2017). Like other MNPs, copper nanoparticles (CuNPs) have also shown excellent antimicrobial activity and changes in the morphology of microbial cell is suggested to be the plausible cause of their biocidal action (Bogdanović et al., 2014). Other examples of antimicrobial MNPs are incorporated in Table 1.

Iron oxide has long been known for its application in the biomedical sector due to its biocompatibility and magnetic property. However, analysis of antibacterial property of reduced iron (Fe^0^) and iron oxide nanoparticles (FeO-NPs) is relatively new. The bactericidal effect of FeO-NPs is observed either due to disruption of cell membrane, or oxidative stress inside the cell, or both (Lee et al., 2008; Arokiyaraj et al., 2013) or due to oxidation of protein and peroxidation of membrane lipids (Dinali et al., 2017). Compatibility of ZnO-NPs with human skin and their safety has made them appropriate additive for cosmetics, fabrics, and surfaces that remain in close proximity of human body (Dizaj et al., 2014). Owing to their microbicidal effect on both Gram positive and Gram negative bacteria, ZnO nanocomposites have been applied in food packing applications (Espitia et al., 2012). The probable mechanisms behind their antimicrobial action are the generation of ROS, the release of Zn ions, and the cell membrane dysfunction (Dizaj et al., 2014). Copper oxide nanoparticles (CuO-NPs) have been exploited for widespread applications, such as gas sensing, batteries, catalysis, etc. In recent past, CuO-NPs were studied for their antimicrobial property and were reported to possess excellent bactericidal and fungicidal activity (Ren et al., 2009). Changes in surface and morphology of microbial cell are supposedly the plausible cause of their biocidal action. TiO_2_-NPs alone and in conjugation with non-toxic polymers exhibit spectacular antimicrobial property. Due to high refractive index and whiteness property TiO_2_-NPs (especially anatase form) have been used in a varied range of consumer merchandises, such as sunscreen lotions, paints, cement, coatings, and toothpaste (Weir et al., 2012). They have also been studied for their potential of potable water disinfection as they are inexpensive, significantly stable in water, nontoxic after ingestion, and result in photocatalytic disinfection (Li et al., 2008). The bactericidal effect of TiO_2_-NPs is strongly related to the formation of ROS, particularly—OH free radicals.

### Fullerenes, graphene, and carbon nanotubes

Not many reports exist on the mode of antimicrobial action of fullerenes (C_60_) and their derivatives thus, it would not be wise to propose their plausible applications. C_60_ and their certain derivatives have shown strong bactericidal activity; however, no such effect is evident in case of fullerols but they have shown virucidal activity. The antimicrobial effect of C_60_ and fullerol is attributed to ROS independent oxidation and formation of highly reactive singlet oxygen species, respectively. The ability of encapsulated fullerene to show antimicrobial effects in water (Lyon et al., 2006) can be used to solve waste water problems. Lately, owing to exclusive surface properties, graphene-based materials like oxides, reduced oxides (rGO), and nanocomposites have caught researchers' attention for their ability to act as antimicrobial agent (Zhu et al., 2017; Jilani et al., 2018); however, only limited number of reports are available in this regard. The mechanism behind their microbicidal activity is mostly accredited to “sheet effect” (Ocsoy et al., 2017), cell membrane dysfunction, and oxidative stress inside the cell (Liu et al., 2011). Depending on their ability to prevent microbial contamination, graphene-based materials have potential to be used in food packaging. Like other aforementioned NSMs, single-walled nanotubes (SWNTs) have also displayed bactericidal activity against both Gram-positive and Gram-negative bacteria, but not much work has been done in this direction. The recognized mode of microbial toxicity behind SWNTs is believed to be either oxidative stress that aborts integrity of cell membrane or their adhesion onto the microbial surface (Dizaj et al., 2014). CNTs have also been used in filters and incorporated into hollow fibers to inhibit bio-fouling of surfaces and formation of biofilms (Li et al., 2008). In addition, they have also been studied for their application as construction material to impart crucial benefits like mechanical durability, crack prevention, biocidal activity, etc. (Lee et al., 2010).

### Nanoscale chitosan (NCH)

NCH as an antimicrobial agent has strong potential for potable water disinfection across membranes or water storage tank surface coatings. Owing to its strong, broad-spectrum microbicidal action and innocuous effect on vertebrate animals, NCH has superseded other disinfectants (Beyth et al., 2015). In recent years, NCH has found its application not only in healthcare and consumer merchandises but also in agriculture and biomedical products (bone cement and wound dressing material), food packaging, waste water treatment, etc. (Li et al., 2008). The exact mechanism behind its microbial toxicity is not very clear; however, loss of cell wall integrity and consequent alteration in membrane permeability has been reported by Kong et al. (2008). Also, electrostatic attraction amid polycationic chitosan and anionic bacterial cell membrane in some cases is known to neutralize and eventually reverse the bacterial cell surface charge. Loss of semi-permeability of the membrane has been suggested to cause intracellular components leakage and ultimately cell death (Kong et al., 2010; Wassel and Khattab, 2017).

### Organic nanoparticles

Although a wide range of antimicrobial drugs is available which can efficiently kill or hamper microbial growth, however, their ineffective and inefficient delivery to the target may result in the poor therapeutic index and cause several local and systemic side effects. In last few years, antimicrobial drugs encapsulated in ONP systems have appeared as path-breaking and promising alternatives that have not only increased therapeutic index but also reduced detrimental side effects of the drug (Yang et al., 2009; Nath and Banerjee, 2013). Currently, liposome is one of the most commonly used antimicrobial drug delivery system because it can mimic the microbial cell membrane and easily fuse with the pathogenic microbe (Pushparaj Selvadoss et al., 2017). Owing to the unhindered fusion of microbial cell membrane and liposome, cargos (drugs) easily get released inside the microbial cell and eventually result in its death (Walsh et al., 2001; Yang et al., 2009). Polymeric nanoparticles (PNPs) have also been extensively studied for their potential to deliver wide variety of antimicrobial agents, as they offer numerous unique features like stable structure, narrow size distribution, zeta potential, ability to finely tune drug release profile, etc. (Cheng et al., 2007; Gu et al., 2008). Like PNPs, dendrimers also possess several exceptional properties, such as large surface area, high *in vivo* reactivity, and ability to load both polar and non-polar agents, which make them a suitable nano-platform for microbicidal drug delivery (Zhang et al., 2010). Not only this, dendrimer itself can act as a powerful microbicide by using the antimicrobial agent as an elementary unit and the plausible mode of microbial toxicity is accredited to the polycationic structural feature which facilitates its adsorption onto the negatively charged bacterial cell. Once adsorbed, increased membrane permeability is witnessed that ensures entry of more dendrimers inside the cell which later facilitate K^+^ ions leakage and complete loss of bacterial membrane integrity (Chen and Cooper, 2002; Ladd et al., 2017). The detailed discussion on antimicrobial activity of dendrimers has been described elsewhere (Scorciapino et al., 2017).

## Limitations of present work and future prospects of aNSMs

The exact mechanism behind antimicrobial effects of NSMs still remains unclear. Certain reports recognize ROS generation or development of oxidative stress as a cause of microbicidal effect, while others suggest antimicrobial effect cannot be associated with metabolism regulation (Dakal et al., 2016). Therefore, addressing exact mechanism behind the antimicrobial action of NSMs should be considered in future work. Several microbes present complex cell membrane structure, therefore, the *in vitro* models cannot completely mimic the *in vivo* conditions to accurately study the effect of aNSMs in duplicate real systems. Other limitations of the current works include lack of unified standards to compare antimicrobial effects of NSMs in order to ensure their potency as antimicrobial agent. Application of NSMs in waste water treatment has raised serious health concerns due to their aggregation in water. Further, loss of nanoparticles during downstream processing may cause toxicity in human beings and affect different ecosystems, therefore future work should be directed toward developing better technologies for retention of nanomaterials. Also, cost effective NSMs should be looked for the disinfection purpose in order to compete with conventional disinfectants.

## Conclusions

Owing to their spectacular properties, NSMs in both organic and inorganic forms have engendered several interesting fields in science and technology. Incessant investigation for their application has led to the development of practical productions and commercialization of products in some cases. Considering the current scenario of human health, its comfort, and well-being; NSMs have been welcomed open-heartedly by several industries, such as health and personal care industry, textile industry, environmental industry, etc. However, realizing the application of NSMs at large scale in the economic setup is still a long shot. Therefore, future work should be directed toward designing novel, applicable, and inexpensive methodologies for scaled up manufacturing of these NSMs in order to meet the growing human needs.

## Author contributions

AB and PC: wrote the manuscript; AS: helped in writing; PK, PM, and VB: edited the manuscript. All authors proofread and finalized the manuscript.

### Conflict of interest statement

The authors declare that the research was conducted in the absence of any commercial or financial relationships that could be construed as a potential conflict of interest.
